# Anti-sulfatide antibodies in neurological disorders: should we test?

**DOI:** 10.1007/s00415-024-12668-8

**Published:** 2024-09-03

**Authors:** Benedict Kleiser, Niklas Giesche, Markus C. Kowarik, Evelyn Dubois, Marcel Armbruster, Alexander Grimm, Justus Marquetand

**Affiliations:** 1 Department of Epileptology, Hertie-Institute for Clinical Brain Research, University of Tübingen, Hoppe-Seyler-Str. 3, 72076 Tubingen, Germany; 2 Department of Neural Dynamics and Magnetoencephalography, Hertie-Institute for Clinical Brain Research, University of Tübingen, Tübingen, Germany; 3 MEG-Center, University of Tübingen, Tübingen, Germany; 4 Department of Neurology and Stroke, Hertie-Institute for Clinical Brain Research, University of Tübingen, Tübingen, Germany; 5 Institute for Modelling and Simulation of Biomechanical Systems, Pfaffenwaldring 5a, 70569 Stuttgart, Germany

**Keywords:** Anti-sulfatide antibodies, Sulfatide, Autoimmune, Neurological disorders

## Abstract

**Objective:**

Neurological autoimmune peripheral and central nervous system disorders can be associated with anti-sulfatide antibodies. These antibodies are considered potential diagnostic biomarkers, although their additional diagnostic value in neurological fields has been increasingly questioned. Given the little evidence of anti-sulfatide antibodies’ frequency and diagnostic value in neurology, we aimed to fill this knowledge gap by investigating 10 years of data.

**Methods:**

This retrospective study analyzed the results of the anti-ganglioside dot kits (GA Generic Assays GmbH) from 1318 serum samples and 462 cerebrospinal fluid (CSF) samples for the frequency, sensitivity, and specificity of anti-sulfatide antibodies in neurological disorders.

**Results:**

Although anti-sulfatide antibodies are rarely present in neurological autoimmune disorders (serum IgM 2.5%, IgG 4.6%), they are also present in non-autoimmune diseases (serum IgM 1.2%, IgG 2.5%) and lack sensitivity and specificity towards being a diagnostic marker. Furthermore, anti-sulfatide antibodies are rarely found in CSF (e.g., no positive results for IgM), and including so-called borderline results ((+)) increases sensitivity and the false-positive rate in serum and CSF.

**Discussion:**

While anti-sulfatide antibodies appear more frequently in neurological autoimmune diseases, they are rare overall and provide very limited diagnostic value in determining specific neurological diseases and—more importantly—if a neurological disease has a potential autoimmune etiology.

## Introduction

Neurological autoimmune diseases of the peripheral [1, 14] and the central nervous system [9] can be associated with anti-sulfatide antibodies, as they target sulfatides primarily found in myelin in the central [8, 12] and peripheral nervous system [4, 12]. While diseases like multiple sclerosis (MS) have other commonly used, clinically established laboratory parameters like oligoclonal bands (OCBs) available as diagnostic biomarkers, this circumstance is not the case for other diagnoses such as autoimmune polyneuropathies. Therefore, the diagnostic value of various antibodies as biomarkers, including ganglioside [7], paranodal/nodal [5], and anti-sulfatide antibodies [1, 14], have been investigated in the past, although in parts, their diagnostic utility is currently increasingly questioned. For example, the added diagnostic value of anti-sulfatide antibodies has been viewed increasingly critically regarding inflammatory forms of polyneuropathy (e.g., [1, 6, 10]).

Nevertheless, in the past decades, their additional diagnostic value in different medical fields has been increasingly questioned due to different studies showing their lack of diagnostic utility in specific neurological disorders. Thus, a comprehensive study is required that provides reference regarding frequency, sensitivity, and specificity for anti-sulfatide antibodies for neurological disorders, both in the two compartments of our nervous systems, i.e., in serum and CSF, as antibodies against sulfatides have been proposed as a potential diagnostic marker for autoimmune processes affecting the myelin sheath of the entire nervous system. To fill this knowledge gap, we aimed to investigate anti-sulfatide antibodies in neurological disorders of the peripheral and central nervous system in serum and CSF and analyze the frequency of anti-sulfatide antibodies based on data from over 10 years of application in our center.

## Methods

### Study design, patients, and procedures

**Fig. 1:**
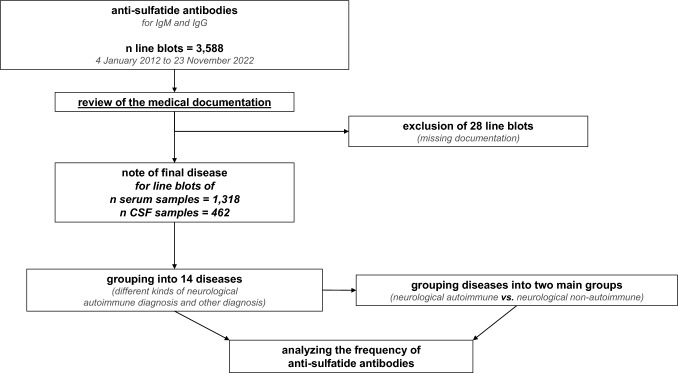
Study workflow. Out of 3588 line blots, 28 were excluded. The remaining line blots, corresponding to 1318 serum samples and 462 CSF samples, were categorized into 14 disorders. These diseases were then grouped into two main categories and analyzed for frequency

1318 serum samples and 462 cerebrospinal fluid (CSF) samples from the neurological laboratory of the University Hospital Tübingen, Tübingen, Germany, collected between January 4, 2012, and November 23, 2022 were analyzed using anti-ganglioside dot kits (GA Generic Assays GmbH) for the evaluation of anti-sulfatide antibodies. Next, each line blot’s result was cross-referenced with the diagnosis documented in the related patient’s medical records. A medical student expert in neurology (NG) reviewed the medical records of all patients, extracting primary neurological diagnoses made during testing when available. He then presented his findings to an experienced neurologist (JM), who eventually confirmed the diagnoses, based on all available clinical documentation (study workflow shown in Fig. 1). Only patients with one test for either serum and/or CSF were included. The ganglioside antibodies included in the test have been previously published elsewhere [7]. The semiquantitative assessment of the color intensity of the blots was categorized according to the manufacturer’s recommendations into negative (−), borderline (( +)), single positive (+), double positive (++), or triple positive (+++). Subsequently, the results categorized as single positive (+), double positive (++), or triple positive (+++) were grouped as positive, and those categorized as negative (−) and borderline ((+)) were grouped as negative. Due to the unclear significance of borderline findings ((+)), the impact of positively counting these findings was also separately evaluated.

Each line blot was then correlated with the disease recorded in the patient’s medical records and sorted into one of 14 disease categories. Whenever feasible, the classification was assigned to a neurological autoimmune-related disease, such as Guillain–Barré syndrome, acute motor axonal neuropathy (AMAN), Miller–Fisher Syndrome, multifocal motor neuropathy (MMN), chronic inflammatory demyelinating polyneuropathy (CIDP), other autoimmune polyneuropathies, MS, or other autoimmune encephalopathies. If the respective disorder did not suggest neurological autoimmune etiology, the probe was sorted into a category of non-autoimmune diseases (other polyneuropathies, amyotrophic lateral sclerosis, other motor neuron diseases, other non-neurological conditions, and cases with no available diagnosis).

### Statistical analyses

Data were analyzed using Statistical Package for the Social Sciences (SPSS) version 29 for Windows, IBM Cooperation, Armonk, NY, USA, and Microsoft Excel 2019 version 1808, Microsoft Office Standard 2019, Redmond, Washington, USA. Figures were also created using Microsoft Excel 2019 version 1808. The absolute and relative frequencies of positive results for each antibody were calculated within each disease group. For further analysis, diseases were categorized into two primary groups: neurological autoimmune-linked and non-autoimmune-linked. Absolute frequency, sensitivity, false-positive rate, and specificity for each antibody were calculated. The statistical association between a positive antibody result and a neurological autoimmune disease was assessed using the Pearson Chi-square test and the phi coefficient (*φ*). Since a borderline ((+)) result is possible, the results are presented by considering these as either negative or positive.

## Results

**Fig. 2:**
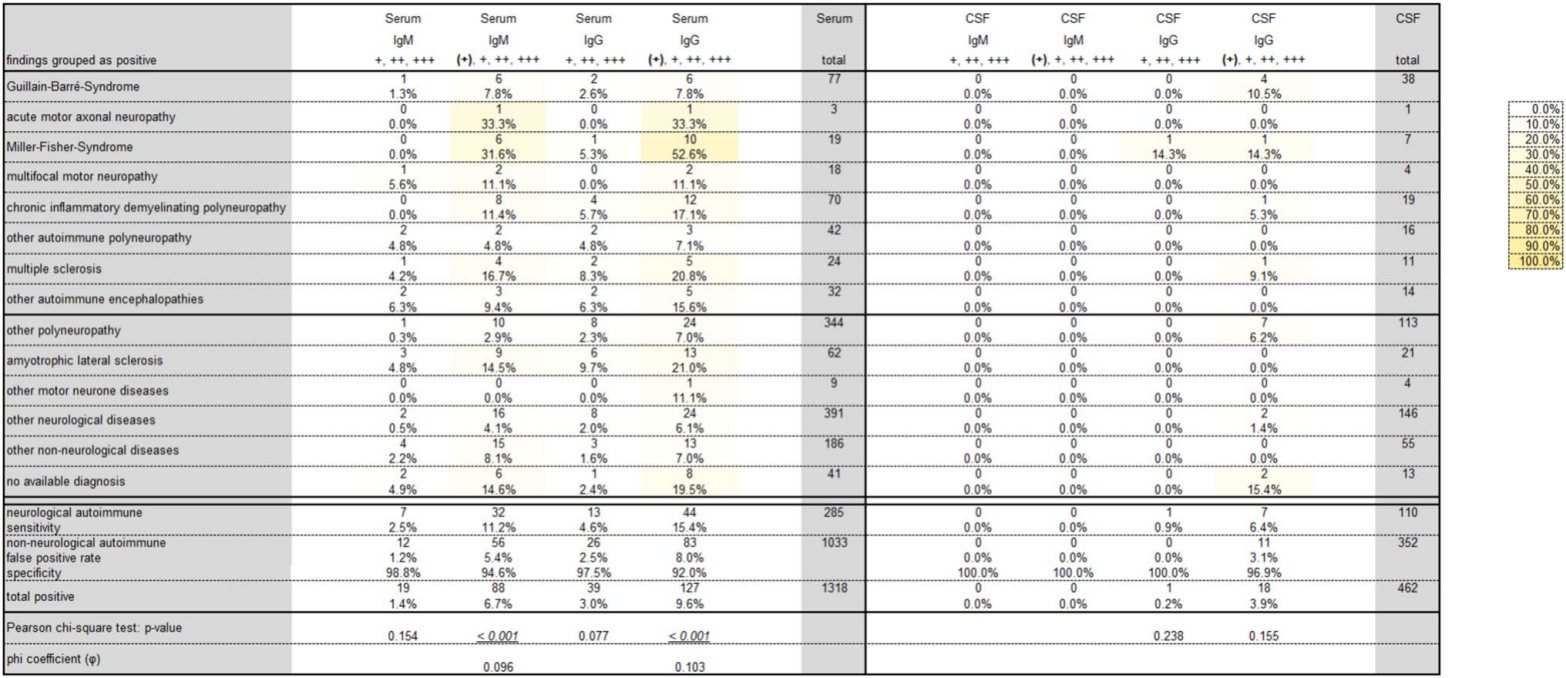
Main results regarding the frequency of anti-sulfatide antibodies. A total of 1318 serum samples and 727 cerebrospinal fluid (CSF) samples were included, with positivity rates calculated for each disorder. Due to the unclear significance of borderline findings ((+)), the impact of positively counting these findings was also separately evaluated. Columns are divided for Serum and CSF as well as IgM and IgG. The total number of performed blots (“total”) per disorder is given at the right for serum and CSF. The relative number of positive results is visually represented using a color scale, where yellow denotes high positive rates and white indicates low positive rates. The rows below summarize positive line blots for the main groups: neurological autoimmune and non-neurological autoimmune, showing sensitivity, false-positive rate, and specificity for each antibody. If Pearson-Chi-square test could be calculated for comparison of neurological autoimmune and non-neurological autoimmune, the *p* values are given. For significant results, the effect size is evaluated by the phi coefficient (*φ*)

A total of 1318 serum samples and 462 CSF samples, each tested with a line blot for IgM and IgG, were included in this analysis (Fig. 1). 28 line blots were excluded due to lack of assignment to a patient or missing medical records. Figure 2 shows the absolute and relative frequencies of positive results for each antibody and disease, along with the absolute frequency, sensitivity, false-positive rate, and specificity for each antibody within the two main groups, for both serum and CSF in IgM and IgG. The sensitivity of individual antibodies ranged from 0.0% to 15.4%, while specificity was consistently above 92%.

However, considering borderline results ((+)) as positive results significantly increased sensitivity (e.g., serum for autoimmune-linked IgM, 2.5% vs. 11.2%, see Fig. 2) while also increasing the false-positive rate (e.g., serum for autoimmune-linked IgM, 1.2% vs. 5.4%). In addition, there was a notable difference in positive findings between CSF and serum (e.g., a total of 6.7% positive, including borderline results for IgM in serum vs. 0.0% for IgM in CSF).

A significant association between the presence of anti-sulfatide antibodies and an autoimmune diagnosis was observed only when borderline results were considered positive in serum for IgM (*p* < 0.001, *φ* = 0.096) and IgG (*p* < 0.001, *φ* = 0.103), which correspond to a small effect size. Otherwise, no significant association was found between anti-sulfatide antibodies and a neurological autoimmune disorder (see Fig. 2).

## Discussion

This study investigated the frequency of anti-sulfatide antibodies by line blots in 1318 serum samples and CSF samples from primarily neurological patients. Our results may mirror real-world clinical settings since the data were derived from routine clinical care. In this context,

1. anti-sulfatide antibodies are more common in the neurological autoimmune disorders than other diseases (e.g., serum IgM 2.5% vs. 1.2%). However, anti-sulfatide antibodies are infrequently positive in the group of neurological autoimmune disorders as well as in other diseases, lacking significance for neurological autoimmune disorders.
2. comparing the frequency of positive results in serum and CSF, anti-sulfatide antibodies are very rarely found in CSF (e.g., no positive results for IgM), leading to very low sensitivity in CSF.
3. adding the borderline results ((+)) to the positive results (+, ++, +++) increases sensitivity but also raises the rate of false-positive results.

### Anti-sulfatide antibodies in serum

In our study, more anti-sulfatide antibodies were detectable in the neurologically autoimmune group, consistent with past studies reporting increased occurrence in autoimmune disorders such as MS [9] or autoimmune polyneuropathy [1, 14]. However, the diagnostic value of these antibodies is increasingly being questioned. In the context of autoimmune peripheral neuropathies, it is noteworthy that they are generally rare, often associated with an IgM monoclonal gammopathy, and found in various forms like CIDP or MMN [1, 6]. In our study, anti-sulfatide antibodies were present in only 2.5% of IgM and 4.6% of IgG in the group of neurological autoimmune disorders. A significant association between the presence of such disorders and anti-sulfatide antibodies was found only when borderline results were considered positive, and even then, the association was very weak. When borderline results were considered negative, no association was found, supporting the critical perspective on the routine use of anti-sulfatide antibodies. However, anti-sulfatide antibodies and the study of sulfatides as biomarkers may play a crucial role in the pathophysiology of neurological autoimmune disorders and might thus contribute to our understanding of autoimmune disorders in the central and peripheral nerve systems [12, 13].

In summary, the measurement of anti-sulfatide antibodies do not seem to provide a clear diagnostic benefit in the context of neurological disorders, although they do contribute to a more comprehensive understanding of autoimmune neurological disorders.

### Anti-sulfatide antibodies in CSF

In our analysis, almost no CSF samples were positive for anti-sulfatide antibodies (only one sample from a patient with Miller–Fisher Syndrome was positive). When borderline results ((+)) were also considered positive, there were still no positive results for IgM, but 18 of the 462 samples were positive for IgG. However, only 7 of these were associated with a neurological autoimmune disease. These results are surprising as they show an even lower frequency of positive antibodies in CSF compared to serum. Notably, for patients with MS, previous studies have reported the presence of anti-sulfatide antibodies in CSF in 15 of 76 samples (19.7%) and discuss it as a part of the intrathecal antibody production [9], while in our study, only 1 of 11 samples from MS patients was borderline positive ((+)) for IgG. This discrepancy could be due to methodological differences between test procedures, as the previous study used ELISA (but only for CSF) [9], while we used a line blot and methodological differences are well-known for other antibodies, such as myositis antibodies [2, 3]. Furthermore, depending on the methods, different anti-sulfatide antibodies and their binding pattern can vary [11].

Overall, given the very low detection rate in 462 samples and the lack of clear diagnostic benefits, determining anti-sulfatide antibodies in CSF can probably be neglected.

### The influence of borderline results

In the line blot used here, a semiquantitative differentiation of intensity can be made into negative (−), borderline ((+)), single positive (+), double positive (++), or triple positive (+++). In clinical practice, the significance of borderline ((+)) results is often questioned. Our study demonstrated that including these borderline results increased the number of positive outcomes for both IgM and IgG in both the autoimmune and non-autoimmune groups, especially in serum samples (e.g., serum for autoimmune-linked IgM, 2.5% vs. 11.2%).

It is known from other antibodies (e.g., myositis antibodies [15]) that low measured intensities in semiquantitative tests can influence results. Similar observations have been made for anti-sulfatide antibodies when measuring titers. In autoimmune polyneuropathies, high titers (> 1/8000) of anti-sulfatide IgM are associated with chronic, typically sensorimotor, autoimmune neuropathy (i.e., CIDP) and the concurrent presence of IgM monoclonal gammopathy in polyneuropathy, while low titers are associated with various neuropathies (1/8000) or show a similar distribution across all patients (1/4000) (0 normal subjects for 1/4000) [1]. Although our study only provides a semiquantitative determination of anti-sulfatide antibody intensity, including borderline findings, which may correspond to low titers, it also led to significantly more positive results in the non-autoimmune patient group. However, it is unclear what actual titer these findings might correspond to, and due to the retrospective nature of this study, exact antibody titers cannot be determined for the presented patient collective.

### Strengths and limitations

This study has several strengths: (a) a large sample size of line blots from predominantly neurological patients, (b) the inclusion of both serum and CSF samples, and (c) the consistent use of an established test system. However, there are some limitations as it is a retrospective study: (a) Patients were classified based on the available clinical information and findings, resulting in more comprehensive documentation for neurological diseases and less for non-neurological diseases. (b) There remains the possibility that patients without a current diagnosis of a neurological autoimmune disease could develop one later. (c) Some disease groups had relatively few patients, with sample sizes as small as three serum samples and even fewer CSF samples, limiting the ability to determine the frequency of antibodies within these groups accurately. For instance, a single positive test result could represent a relatively high percentage, such as 33.3%. In addition, more samples were required for various types of polyneuropathy compared to different autoimmune encephalitis, leading to differing levels of informativeness across disease groups. (d) The test used provides only semiquantitative results, whereas exact results obtained by measuring titers could be more useful for classifying the results.

## Conclusion

Although anti-sulfatide antibodies appear more frequently in neurological autoimmune disorders, they are rare overall and provide a very limited diagnostic value in determining whether a neurological disease is autoimmune. Overall, anti-sulfatide antibodies are detected much more frequently in serum than in CSF, and adding borderline results ((+)) to the positive results (+, ++, +++) is associated with higher sensitivity but also raises the rate of false-positive results.

## Data Availability

Anonymized data will be made available by reasonable request from any qualified investigator.

## Abbreviations

AMAN: Acute motor axonal neuropathy
CIDP: Chronic inflammatory demyelinating polyneuropathy
CSF: Cerebrospinal fluid
MMN: Multifocal motor neuropathy
MS: Multiple sclerosis
OCBs: Oligoclonal bands

## Declarations

### Standard protocol approvals, registrations, and patient consents

The study was performed according to the Declaration of Helsinki and was approved by the local ethics committee (project number approval no. 189/2023B O2, 25th April 2023).

### Declaration of generative artificial intelligence and artificial intelligence-assisted technologies in the writing process

After writing the draft of our manuscript, we utilized Large Language Models (LLMs, here ChatGPT and Grammarly) to correct for grammar and scientific rigor. After using this tool, the authors reviewed and edited the content as needed.
